# Epigenetic modulation in obsessive-compulsive disorder: Methylation and hydroxymethylation of the bdnf gene exon I promoter

**DOI:** 10.1192/j.eurpsy.2021.348

**Published:** 2021-08-13

**Authors:** S. Vanzetto, V. Alberto, D. Conti, M. Macellaro, B. Benatti, C. Daddario, F. Bellia, B. Dell’Osso

**Affiliations:** 1 Luigi Sacco Hospital, Psychiatry 2 Unit, University of Milan, Milan, Italy; 2 Department Of Clinical Neuroscience, Karolinska Institutet, Stockholm, Sweden; 3 Department Of Bioscience, University of Teramo, Teramo, Italy; 4 “centro Per Lo Studio Dei Meccanismi Molecolari Alla Base Delle Patologie Neuro-psico-geriatriche”, University of Milan, Milan, Italy; 5 “aldo Ravelli” Center For Nanotechnology And Neurostimulation, University of Milan, Milan, Italy; 6 Department Of Psychiatry And Behavioral Sciences, Stanford University, Stanford, California, United States of America

**Keywords:** Molecular Neurobiology, genetics, Obsessive-Compulsive disorder, Neuroscience in Psychiatry

## Abstract

**Introduction:**

Several evidence recognizes Brain Derived Neurotrophic Factor (BDNF) as a promising biomarker in the pathophysiology of psychiatric disorders, including Obsessive-Compulsive Disorder (OCD), considering the involvement of epigenetic regulation in BDNF altered expression.

**Objectives:**

This study aims to investigate, in a sample of OCD patients, the epigenetic modulation in terms of levels of methylation and hydroxymethylation on the BDNF gene exon I promoter.

**Methods:**

Fifty OCD patients, recruited from Psychiatry Unit 2, Sacco University Hospital in Milan and fifty healthy controls, comparable by age and gender. Saliva samples were collected by oral swab and epigenetic analysis were performed at the University of Teramo. Statistical analyses were performed with t test with Bonferroni correction.

**Results:**

Data analysis showed a significant decrease in 5-methyl cytosine levels (5mC) (mean OCD: 1.221%; mean CTRL: 1.784%; p < 0.001) and a significant increase in 5-Hydroxy-methyl cytosine levels (5hmC) (mean OCD: 1.018%; mean CTRL: 0.527% p< 0.0001) in BDNF gene exon I promoter of OCD patients compared to controls. Regarding 5mC of site 3 and 5hmC of site 1 and 2 of the exon I promoter CpG islands, no statistical significance was found.

**Conclusions:**

Present results showed significant differences in epigenetic modulation of BDNF gene, which might not be univocally interpreted. They could represent an intrinsic OCD characteristic or the effect of antidepressant drugs, assumed by all recruited patients. Further studies, comparing OCD subjects in treatment vs drug-free, are necessary to define BDNF epigenetic modulation role and its possible use as biomarker in the characterization of OCD.

**Disclosure:**

No significant relationships.

